# Bridging the gap between reference and real transcriptomes

**DOI:** 10.1186/s13059-019-1710-7

**Published:** 2019-06-03

**Authors:** Antonin Morillon, Daniel Gautheret

**Affiliations:** 1 ncRNA, Epigenetic and Genome Fluidity, CNRS UMR 3244, Sorbonne Université, PSL University, Institut Curie, Centre de Recherche, 26 rue d’Ulm, 75248 Paris, France; 2 Institute for Integrative Biology of the Cell, CEA, CNRS, Université Paris-Sud, Université Paris Saclay, Gif sur Yvette, France

## Abstract

Genetic, transcriptional, and post-transcriptional variations shape the transcriptome of individual cells, rendering establishing an exhaustive set of reference RNAs a complicated matter. Current reference transcriptomes, which are based on carefully curated transcripts, are lagging behind the extensive RNA variation revealed by massively parallel sequencing. Much may be missed by ignoring this unreferenced RNA diversity. There is plentiful evidence for non-reference transcripts with important phenotypic effects. Although reference transcriptomes are inestimable for gene expression analysis, they may turn limiting in important medical applications. We discuss computational strategies for retrieving hidden transcript diversity.

**Electronic supplementary material:**

The online version of this article (10.1186/s13059-019-1710-7) contains supplementary material, which is available to authorized users.

## Reference transcriptomes: the making of

Reference transcriptomes (RefTs) aim to provide a comprehensive picture of transcripts produced by an organism. Early RefTs were produced at the turn of the century based on sanger sequencing of full-length cDNAs (flcDNA) [1–3]. Later on, projects such as ENCODE, modENCODE, and FANTOM5 harnessed the power of massively parallel cDNA sequencing (RNA-seq) to accelerate transcript discovery in multiple species and tissues. Due to limited RNA-seq read size (approximately 100 nucleotides), these efforts had to include additional technologies to guarantee accurate full-length transcript assembly. For instance, the FANTOM5 RNA-seq based human cDNA collection was assembled with assistance of the CAGE technology to identify RNA 5′ ends, ENCODE transcript sets were based on RNA-seq and rapid amplification of cDNA ends (RACE) technologies [4], and the fly and *Caenorhabditis elegans* ModENCODE sets combined RNA-seq, RACE, and expressed sequence tag (EST) sequencing [5, 6]. In yeast, major transcriptomics efforts have involved CAGE, TIF-seq, high coverage paired-end RNA-seq (both total and poly(A)+) and 3′-end tags, covering both stable and cryptic transcripts [7–10] . A third generation of transcriptomics projects now combines single-molecule, long-read sequencing technologies with short-read sequencing. Long-read-based datasets are now available for human [11, 12] and several plants [13, 14] and new sets of high-quality full-length transcripts are expected for all model species

**Fig. 1:**
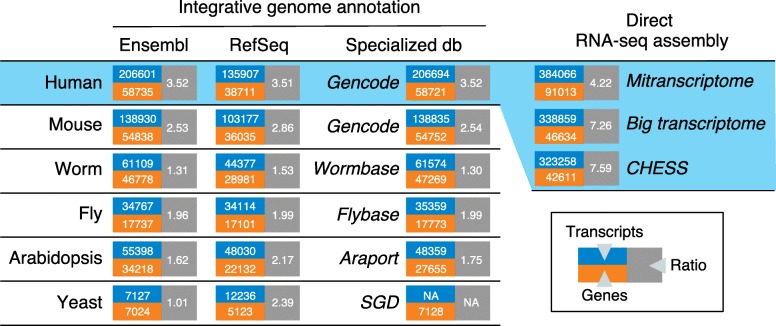
Contents of major reference transcriptomes for human and model eukaryotes. Versions of databases used: Ensembl [15], V95; RefSeq [16], human rel 109, mouse rel 106, worm rel WS268, fly rel 6.18, arabidopsis rel TAIR10.1, yeast rel R64–2-1; Gencode [17], Human V29, mouse M20; Wormbase [18], WS268; Flybase [19], r6.26; Araport [20], V11; Saccharomyces Genome Database (*SGD*) [21], V20150113. Database URLs and additional curation information are provided in Additional file 1: Table S1

Major genome databases integrate sequence data from the above sources into non-redundant, curated transcript datasets (Fig. 1). RefSeq [16] and Ensembl [15] are pan-species databases that implement a homogenous computational annotation workflow combining assembled high-throughput data and manually curated transcripts when available. Specialized RefTs such as Gencode for human and mouse [17, 22], Wormbase for *C. elegans* [18], Flybase for *Drosophila* [19, 23], and Araport for *Arabidopsis* [20], are produced through a combination of manual curation of full-length transcript collections from various origins and dedicated short-read assembly software. The Saccharomyces Genome Database [21] does not provide a set of full-length transcript sequences; however, RefSeq and Ensembl provide RefTs for yeast.

The most striking lessons drawn from large-scale transcript sequencing have been the widespread expression of long non-coding RNA genes and the abundance of alternative transcripts. This is well reflected in the number of genes and transcripts in current genome annotations (Fig. 1). For instance the human Gencode RefT now harbors 58,721 genes (that is, three times more than coding genes) and a transcript-to-gene ratio of 3.52.

## Enter direct RNA-seq assembly

While current transcript counts in RefTs may seem impressive, these datasets have actually grown relatively slowly, constrained by their rigorous curation process. For instance, Gencode has grown from 161,000 human transcripts in 2012 to 207,000 now, i.e., a 29% growth in 7 years. In the meantime, projects generating raw RNA-seq data have exploded. Three projects alone, The Cancer Genome Atlas (TCGA) [24], GTEX [25], and Human Protein Atlas (HPA) [26], have produced 31,000 RNA-seq libraries covering normal and cancerous tissues from thousands of individuals (Additional file 1: Table S2). Raw RNA-seq datasets have been reanalyzed by direct RNA-seq assembly projects such as miTranscriptome [27], BigTranscriptome [28], and CHESS [29]. These computational protocols, which do not implement the strict validation process used for RefTs, led to a 55–85% growth of the number of annotated human transcripts (Fig. 1; Additional file 1: Table S1). Nevertheless, the largest sets used in direct computational assembly are still 40 times smaller than public RNA-seq databases (over 400,000 human libraries in SRA [30] and ENA [31]). This vast wealth of RNA-seq data contains extensive transcript variation that is not yet included in RefTs. Therefore, a deep information gap may be building up between slow moving RefTs and yet undiscovered RNA variants from short read data.

We describe below the different types of transcript variations that may be missing from RefTs. We contend that the information gap between RefTs and high-throughput data is not going to be closed. Based on multiple evidence gathered from medical transcriptome studies, we argue that non-reference transcript information is highly significant and its neglect limits our understanding of genotype–phenotype relationships. This underlines the need for computational methods that can extract non-reference events from RNA-seq data.

## Shall we ever reach a complete reference transcriptome?

**Fig. 2:**
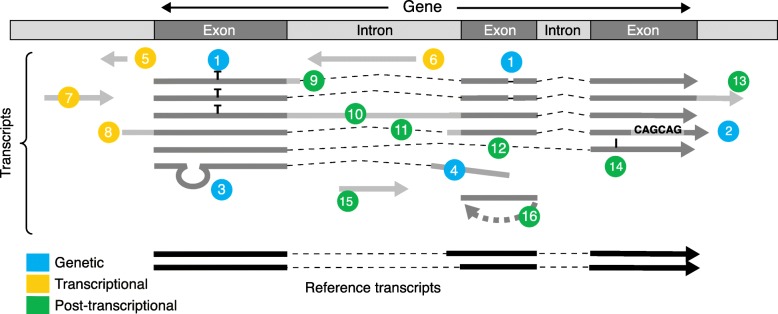
The sources of transcript diversity shown on a typical eukaryotic gene. Genetic: *1* single nucleotide variation or short indel, *2* microsatellite variation, *3* transposition, *4* gene fusion. Transcriptional: *5* bidirectional transcription start site (TSS), *6* antisense transcript, *7* enhancer RNA, *8* alternative TSS. Post-transcriptional: *9* alternative 5′ splice site (SS), *10* alternative 3′ SS, *11* alternative 3′ SS, *12* skipped exon, *13* alternative poly(A) site, *14* editing and modification, *15* processed pre mi/snoRNA, *16* circular RNA

Each cell of an organism produces a distinct set of transcripts. Transcriptome differences between cells stem from three mechanisms that are potentially cumulative (Fig. 2). First, genetic variation occurs across individuals in a population as well as within each individual through aging and cancer. This includes a vast range of variation, from single nucleotide substitutions and indels to mobile element insertion and large chromosomal rearrangements. Second, transcriptional regulation programs are implemented during organism development and cell differentiation. These comprise all variations of transcription activity, whether in intensity, start site, or strandedness. Third, post-transcriptional regulations**,** including a wide array of RNA processing, editing, base modification, and cleavage/degradation mechanisms, are specific to cell type, cell compartment (e.g., splicing in the nucleus), and environmental conditions. It is worthy to note that transcriptomic complexity is not limited to higher eukaryotes, as illustrated by the discovery of bidirectional promoters [9, 32] and cryptic transcripts [7] in yeast.

Most individual RNA variations do not find their way into RefTs. An analysis of splice junctions in approximately 21,500 human RNA-seq libraries from SRA [33] identified over three million junctions supported by at least 20 reads, which is nine times more than found in Gencode transcripts. Yet, the analysis did not include the restricted access TCGA [24] dataset. Considering the importance of aberrant splicing in cancer [34] and other diseases [35], one may expect RNA-seq data from pathological samples to yield large quantities of novel variations. National medical genomics projects will deliver millions more individual sequence sets, including RNA-seq, raising the question of whether these data should eventually be incorporated into RefTs.

One last important factor limiting RefT completeness stems from the nature of RNA libraries analyzed (Additional file 1: Table S3). RefTs are based primarily on poly(A) + libraries, which are far from encompassing all transcripts and present quantitative and qualitative bias related to poly(A) retention efficiency [36]. Alternative RNA selection protocols, including ribo-depleted RNA-seq, nascent RNA-seq, capture-seq, small RNA-seq, M6A-seq, and compartment-specific RNA-seq [37–40], have already revealed large quantities of previously hidden RNAs. The ability to sequence modified RNA bases will add yet another dimension to transcriptomics. As RNA modifications cause abortive reverse transcription, specific protocols are needed to either allow bypass of modified bases or recovery of aborted cDNAs [41]. Alternative strategies involving direct sequencing of modified RNA with the Nanopore technology are still under development.

The above observations are in line with recent studies that have underlined the difficulty of ever completing a mammalian transcriptome. Uszczynska-Ratajczak et al. [42] showed large-scale lncRNAs catalogues are far from converging while Deveson et al. [43] conclude from their analysis of alternative splicing of non-coding exons that “there does not exist a finite list of noncoding isoforms that can be feasibly catalogued”.

## Ignore non-reference transcripts at your own risks

It may be argued that non-reference transcripts are predominantly transient or expressed at a low level and therefore can be ignored as transcriptional [44] or splicing [45, 46] noise. The function of pervasive, intergenic transcripts has been particularly disputed on this basis [47–49]. Although pervasive transcription is now recognized as a source of de novo gene birth [50, 51] and thus may be important for a species as a whole, it is obviously difficult to speculate or raise much interest about future gene functions. A more sensible approach to establish function is arguably that taken by evolutionary biologists who use negative selection as an evidence for function. Selection measures based either on phylogenetic conservation [52] or allele frequencies in populations [53] are converging towards 4–9% of the human genome under selection, which is to be compared with the 1.5% coding fraction. Predicted functional regions include about 130 Mb which are either expressed (mRNA and lncRNA exons and introns) or potentially expressed (enhancers, transposable elements, pseudogenes) [52]. One can reasonably propose that any transcript variation altering these regions, whether genetic, transcriptional, or post-transcriptional, may impact phenotype.

An alternative way to appreciate the biological impact of non-reference transcripts is to consider transcript alterations in human diseases. The list of disease-causing or disease-related transcripts that are not part of the RefT is a long one (Additional file 1: Table S2). Chimeric transcripts [54] and viral transcripts from integrated or free virus, such as human papillomavirus (HPV) [55], are important cancer drivers which are not included in RefTs. Aberrant splicing is a source of key drivers in cancer [56] and other diseases [35, 57]. Alternative polyadenylation events contribute to human disease and are connected with development, cell differentiation, and proliferation [58]. Intron retention events are considered as novel disease factors [59, 60]. Reactivated transposable elements and retrotransposed mRNAs are involved in tumorigenesis [61] and Alzheimer’s disease [62]. Rearranged T-cell receptor transcripts are used to monitor T-cell clonal expansion in tumors [63]. Both A-to-I RNA editing events and M6A base modifications contribute to cancer progression [37]. Two abundant classes of non-reference RNAs, circular and antisense RNAs, have been involved in gene regulation [64] and used as disease biomarkers [65]. Lastly, genetic polymorphism in transcripts, whether in the form of single-nucleotide variants, short indels, or microsatellite expansion, may strongly impact RNA processing, stability, and translation. An extreme illustration is the CAG repeat expansion in the HD gene at the origin of Huntington’s disease [66]. Although sequence polymorphisms are generally ignored in transcriptome studies, taking into account this dimension should lead to a better understanding of the potential impact of transcripts on phenotypes, as the medical community enters the “personal transcriptome” era [35, 67].

## RNA-seq analysis in the personal transcriptome era

RNA-seq data analysis commonly involves mapping reads to an annotated genome or a RefT to quantify transcript and gene expression [68]. These protocols do not permit detection of novel transcripts and may lead to inaccurate expression measures due to incomplete transcript annotations [69]. A straightforward improvement to quantification protocols is to replace a RefT with an extended catalogue generated by direct RNA-seq assembly, as available for human [27–29]. This may work satisfyingly when studying datasets similar to those from which the catalogue originated (TCGA, GTEX, etc.). However, these catalogues have shown large divergences [42] and thus do not guarantee that events present in an arbitrary RNA-seq experiment are covered. The only way to ensure this is to implement a RefT-free strategy.

**Fig. 3:**
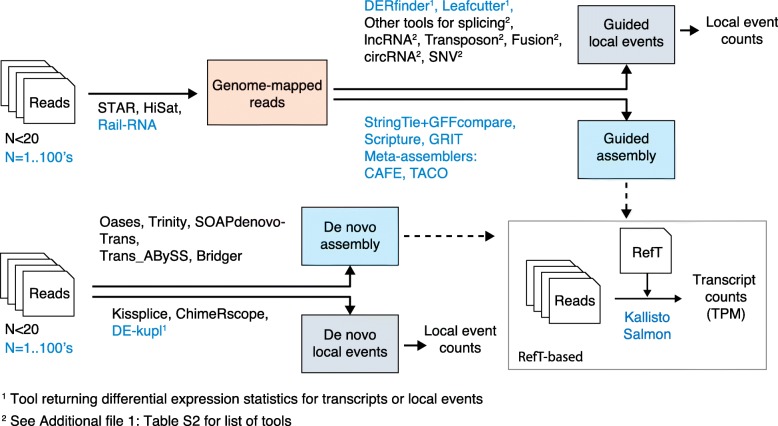
RNA-seq pipelines for the discovery and quantification of transcripts and processing events, unconstrained by a RefT. Software in *black* are best suited to “small” input datasets (represented by an arbitrary size *N* < 20) while software in *blue* can process large datasets (up to hundreds of libraries). Protocols are subdivided into four combinations of genome-guided versus de novo and assembly-based versus local event discovery. Local events include splice variants, transcribed regions, gene fusions, circular RNAs, sequence polymorphisms (*SNV*) and expressed transposons (Additional file 1: Table S2). Results from assembly software can be used as RefTs in standard quantification pipelines (*inset*)

Figure 3 presents a selection of RefT-free software pipelines for RNA-seq analysis. As a guide for users, the figure shows whether pipelines are limited to small numbers of initial libraries (here arbitrarily shown as < 20) or can scale to hundreds of libraries. Two other highlighted differences between strategies are (i) whether or not they attempt full-length transcript assembly and (ii) whether they are genome-guided or de novo methods.

Assembly software predict full-length transcripts either de novo from raw RNA-seq data [70–72] or following genome alignment [73–76]. Major motivations for using assembly software are transcript quantification and analysis of protein-coding potential. De novo assembly is computationally demanding and is mostly used with small datasets and when a reference genome is unavailable. On the other hand, genome-guided assemblers can be applied iteratively to hundreds of RNA-seq libraries. However, a major limitation in all assembly processes stems for their reliance on splicing graph analysis, which has a relatively high error rate that grows with the number of reads analyzed [77–79]. As said by Hayer et al. [78], “with more reads, most algorithms find more ways to go wrong”. The assembly of large datasets is thus performed stepwise, first by assembling individual libraries and then using meta assemblers [28, 29, 80] to merge results. Of note, some assembly protocols are able to use transcript boundary information from CAGE and 3′-seq data to improve assembly quality [76, 80].

Transcript assembly is not the most adequate route in many situations. First, individual transcript variations such as alternative transcription start sites and splicing/polyadenylation events are under-represented in predicted full-length transcripts [81]. Second, assembled transcripts are especially unreliable with certain RNA classes such as the weakly expressed, highly heterogeneous lncRNAs [82]. Third, certain RNAs, such as fusion or circular RNAs, are generally absent from genome-guided assemblies. Therefore, non-canonical or alternative transcription is often best studied using strategies that bypass assembly altogether and focus solely on specific variations recovered from the genome mapping (BAM) files. This category includes powerful software such as LeafCutter [83] for splice site discovery and DERfinder [84] for the characterization of lncRNAs and alternative mRNA boundaries. Other software tools are able to use partly mapped or unmapped reads for the recovery of gene fusions, circular RNAs, single-nucleotide variants, and expressed transposons (Fig. 3; Additional file 1: Table S4).

Genome-guided procedures assume that all samples under study have the same genetic makeup. This does not hold when RNA-seq data come from individuals with significant genetic divergences or from samples harboring somatic structural variations. Transcripts expressed from variable regions may erroneously map to the reference genome, leading to incorrect transcript assemblies and counts. An emerging class of software, including Kissplice [85], ChimerScope [86], and DE-kupl [87], avoid both genome alignment and transcript reconstruction through direct mining of the k-mer (subsequence of fixed size) contents of the original sequence files. These are promising approaches that apply particularly to cases where a reference genome cannot be relied upon.

## Concluding remarks

In spite of continuous updates, RefTs are not catching up on short-read RNA-seq data in their coverage of transcript diversity. Single molecule (long-read) RNA sequencing will help improving RefTs faster than current technologies that require capture of cDNA ends in complement to short reads. However, the combinatorial nature of transcript variation, the higher yield of short-read sequencing, and the huge diversity of tissues, diseases, and transcript classes probed by short-read sequencing make it unlikely that RefTs will ever match the level of diversity observed in short read data.

Of note, limitations of RefTs are in a large part intentional. Indeed, these databases are manually curated to exclude a majority of pervasive transcripts resulting from expressed repeats, pseudogenes, or erroneous splicing. Transcript catalogues computationally generated from thousands of RNA-seq libraries apply less stringent inclusion criteria and are poised to include a large fraction of non-functional and pathological products, as well as incorrect boundaries and exon structures [11, 77].

Well-curated RefTs are essential resources for measuring gene expression. RefT-based gene expression analyzes are now highly efficient [88, 89], provide accurate gene expression measures [90], and can be functionally interpreted via multiple resources for gene ontology and pathway analysis. For these reasons, RefTs will remain a major tool for transcriptomics. Functional analysis of non-reference transcripts is more hazardous as many are non-coding and there is no commonly accepted way to annotate their function. Yet, their impact should not be underestimated. The aforementioned examples taken from human diseases reveal a wide diversity of non-reference transcripts with phenotypic effects. Even though these transcripts might be of low abundance, they can be essential in understanding genotype–phenotype relationships and should not be ignored.

There is no consensus on the most efficient RNA-seq analysis protocols for characterizing and quantifying non-reference transcripts. Strategies focusing on local or regional transcript variations are a powerful way to circumvent limitations related to full-length assembly. Such methods can be combined to conventional RefT-based analysis to achieve a complete description of normal and aberrant transcript forms present in a set of RNA-seq libraries.

## Additional file

Additional file 1: **Table S1.** Overview of major eukaryotic transcriptome databases. **Table S2.** Large-scale RNA-seq projects (human). **Table S3.** Sequencing methods providing insight on specific events shown in Fig 2. **Table S4.** Transcript variations related to cancer and other diseases; and software for retrieving these variations from RNA-seq data. (XLSX 18 kb)

## Abbreviation

RefT: reference transcriptome
